# Effects of Platelet-Rich Plasma on Cartilage Grafts in Rabbits as an Animal Model

**Published:** 2012-07

**Authors:** Ali Manafi, Kamran Kaviani far, Masoud Moradi, Amir Manafi, Farzad Manafi

**Affiliations:** 1 Department of Plastic Surgery, Tehran University of Medical Sciences, Tehran, Iran; 2 Researcher, Medical Student, Tehran University of Medical Sciences, Tehran, Iran; 3 Researcher, Medical Student, Shahid Beheshti University of Medical Sciences, Tehran, Iran

**Keywords:** Cartilage, Graft, Platelet-rich plasma, Regeneration, Rabbit

## Abstract

**BACKGROUND:**

Cartilage tissue has limited regenerative capacity and the management of cartilage defects has always been a challenging issue. Platelet-rich plasma (PRP) has been recently been used to improve healing of cartilage defects. In the present experiment, we aimed to investigate the effects of PRP on regeneration capacity as well as survival of the cartilage grafts in a rabbit model.

**METHODS:**

In 15 white New Zealand rabbits, auricular cartilage was used to produce 4 pieces of cartilage (each about 2×2 cm). Two samples were diced to small pieces and 2 samples left intact. PRP was prepared using 5 ml of auricular blood. After measuring of the weight and volume of cartilages, they mixed with either normal saline or PRP and then implanted on the back of the rabbit. After 12 weeks, the implants were removed and the weight and volume were measured and the numbers of nucleated lacunae were counted on H & E staining.

**RESULTS:**

Histological findings showed that in both the intact and diced cartilages, adding PRP resulted in increasing regeneration of chondrocytes. Moreover, adding PRP to intact cartilages had a significant effect in maintaining the grafts ‘weight and volume (*p*<0.05) but in diced cartilages, this effect was not significant.

**CONCLUSION:**

Our findings yielded valuable information on the effect of PRP on survival and regeneration of autologous cartilage grafts as the extent of angiogenesis and the diameters of vessels were more pronounced in the side using PRP and markedly lesser reduction of weight and volume were visible in this group. PRP was effective in increasing survival and regeneration capacity of cartilage grafts in rabbit model.

## INTRODUCTION

Cartilage regeneration is required in orthopedic, head and neck surgery for the repair of cartilage defects in joints, nose, or auricle. Cartilage tissue has limited regenerative ability, mainly due to its avascular nature.^1^,^2^ As a result, management of cartilage defects has always been a challenging issue for the physicians.

Several attempts have been undertaken to improve the regeneration capacity of cartilage tissue.^3^,^4^ Platelet-rich plasma (PRP) is a highly concentrated form of platelets. Use of autologous PRP is a possible strategy to enhance the wound-healing cascade in both soft and hard tissues. Activation of the platelets of PRP leads to release of conspicuous amounts of various growth factors including platelet-derived growth factor (PDGF), transforming growth factor (TGF), platelet derived angiogenesis factor (PDAF), vascular endothelial growth factor (VEGF), insulin-like growth factor (IGF)-I, platelet-factor (PF-4) and epidermal growth factor (EGF).^5^-^9^

PRP already has been used to promote bone formation at the site of fracture or grafting and also to accelerate the healing of chronic non-healing wounds (10-13). However still data are lacking regarding the effects of on cartilage regenerative capacity. In the present study we aimed to assay the effects of adding PRP to cartilage grafts on their resorption as well as regeneration in a rabbit model.

## MATERIALS AND METHODS

The surgical procedure and care of rabbits were performed under the regulation of Experimental Animal Centre, Fourth Military Medical University, Xi’an, PR China and the study was approved by the Institutional Ethics Review Committee for Animal Research at Tehran University of Medical Sciences. Fifteen young New Zealand male rabbits, each with 5 months of age and weighting between 2 to 2.5 kg, were included in this experimental study. To reduce the impact of transportation stress all rabbits were taken to the lab five day before the surgical procedure.

All animals received general anesthesia using a combination of ketamine (15 mg/kg, intramuscular) and xylazine hydrochloride (5 mg/kg, intramuscular). All rabbits received 60 mg/kg intramuscular ceftriaxone. After induction of anesthesia, 5 ml of blood was with-drawn via ear central vein aspiration and mixed with 3.8% sodium citrate at a ratio of 1 ml sodium citrate to 5 ml of whole blood. This blood was used for preparation of PRP. In cases with unsuccessful auricular blood sampling, intracardiac method was used. As using this method provides more blood volume, we decided to aspirate 10 ml blood in these cases. Five milliliter of this blood was used for preparation of PRP and the remaining 5 ml was saved for determination of platelet concentration of PRP. Finally, blood sampling was carried out via auricular vein in 12 rabbits, and in the remaining three rabbit intracardiac method was used. As two rabbits were kept in a single cage to facilitate the recognition of the subjects in one of them, the left ear and in the other one right ear was used for blood sampling.

The preparation of PRP was performed simultaneously during the surgical procedures. PRP was prepared as previously described.^11^ Briefly, 5 ml of blood was used to prepare PRP for each animal. As mentioned above, solution of 3.8% sodium citrate was used to prevent coagulation. This resulted in a red and opaque lower fraction consisting of red and white blood cells and platelets, which is named the blood cell component (BCC); and a upper yellow turbid fraction containing plasma and platelets, which is called the serum component (SEC). The entire SEC and the upper 6 to 8 mm of BCC is pipetted into a sterile vacurette without citrate. This material is again centrifuged at 2000 rpm for 5 minutes. The top yellow SEC (i.e. platelet poor plasma, PPP) is removed. Finally, the remaining substance is the concentrated platelet i.e. platelet-rich plasma (PRP).

The ear which was used for blood sampling was cut and its stump was sutured by nylon suture 4-0. Then, the skin and perichondrium was removed from the auricular cartilage. The prepared cartilage was divided into 4 pieces with dimensions of 2×2 cm. Two pieces of cartilages were diced to pieces of 0.5-1 mm using the surgical blade number 11 (2 diced cartilages) whereas the other 2 pieces remained intact (2 intact cartilages). Thereafter, all 4 cartilages (2 diced and 2 intact cartilages) were weighted on a scale of 0.01 g sensitivity.

The intact cartilage was inserted into an insulin syringe, and then by using another insulin syringe, 1 ml normal saline was added to it to reach volume of 1 ml. The volume of the remained saline in the second saline was considered as the volume of the cartilage. For determination of volume of diced cartilages, the pieces of cartilages were inserted in the insulin syringe and after mild plunger compression, the volume was measured. To one of the diced cartilages PRP, and to the other one, normal saline was added. Intact cartilages were prepared likewise.

In the back of each rabbit, 2 packets in the right side and 2 packets in the left side were incised with distance of 3 cm from each other between the panniculus carnosus and deep fascia layers. The size of packets was considered as 2.5×2.5 cm for intact cartilages and 1.5×1.5 cm for diced cartilages.

In all rabbits, cartilages were implanted with a constant sequence. In the right side, cartilages without PRP and in the left side, cartilages with PRP were inserted. Intact cartilages were implanted in the cephalic end and diced cartilages in the caudal end.

To determine the platelet concentration, in 3 rabbits which blood sampling was done with intracardiac method, 10 ml blood was taken and divided into two equal 5 ml volumes and two samples of PRP were prepared. One of them was used for mixing with cartilages and the other one was used for assessment of numbers of platelets in the PPP and PRP.

With considering the difference of size of platelets in rabbit and humans, it was possible to make wrong by using the laboratory machines for counting the platelets as a result, counting was done manually by using light microscope. After relative awareness, rabbits were transferred to their cages and for five days, suspension of co-amoxiclav was mixed with their water. To the end of the study, the animals remained in the laboratory research housing facility, receiving water, food and medical care as needed.

**Fig. 1:**
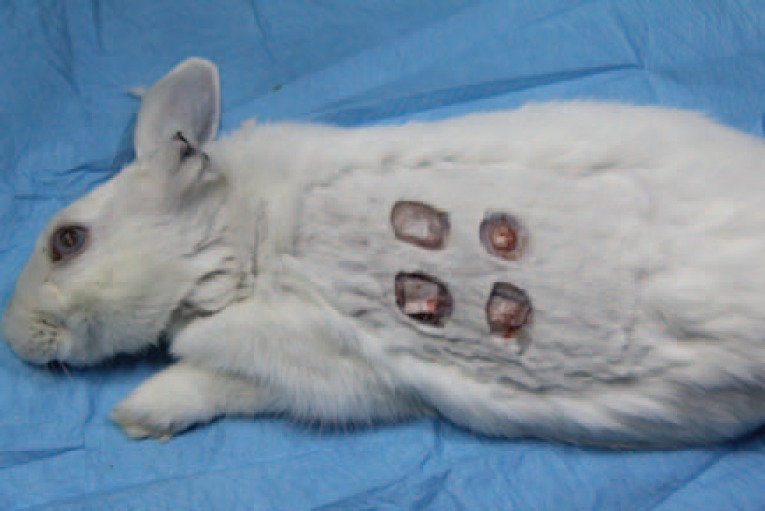
Four packets were incised in the back of each rabbit and four types of cartilage grafts (intact cartilage without PRP, intact cartilage with PRP, diced cartilage without PRP and diced cartilage with PRP) were implanted. PRP: platelet-rich plasma.

After 12 weeks, all rabbits were sacrificed with carbon monoxide and the skin overlying cartilage grafts were removed (Figure 1). All cartilages were harvested, weighted and their volume was recorded. Cartilages were sent to the pathology laboratory in a solution of 10% formaldehyde. After preparing paraffin blocks with 5 µm thick longitudinal serial sections, H & E staining was performed and viability of cartilages was determined by counting the numbers of nucleated lacunae. In each of cartilage samples, the numbers of nucleated lacunae were counted in three separate fields with magnification of ×40 and mean of them was recorded for that sample.

Data were analyzed using SPSS software (Version 19, SPSS Inc., Chicago, IL, USA). Data were represented as mean±SD. All data were assessed for normality and all were normally distributed. For comparing the quantitative variables, one way ANOVA was used. For assessing the differences between groups, Tukey Post Hoc was used. A *P *value of less than 0.05 was considered statistically significant.

## RESULTS

Two rabbits in the first day and one rabbit in the second day following surgery died. The first two rabbits were among those who were bled by intra-cardiac method. No sign of infection was seen in the site of surgery in dead rabbits. Twelve rabbits remained alive by the end of the study. In none of these rabbits, signs of infection or adverse outcomes of surgery were seen.

Considering the adherence to the surrounding structures, intact cartilages had lesser adherence and their harvesting was performed more easily. No significant difference was seen between cartilages with and without PRP in this regard. Adherence of diced cartilages to the surrounding structures was more pronounced and their harvesting needed more dissection. In this group, no difference was seen between cartilages with and without PRP. Diced cartilages in both groups (with and without PRP) have been strongly adhered to each other.

**Fig. 2:**
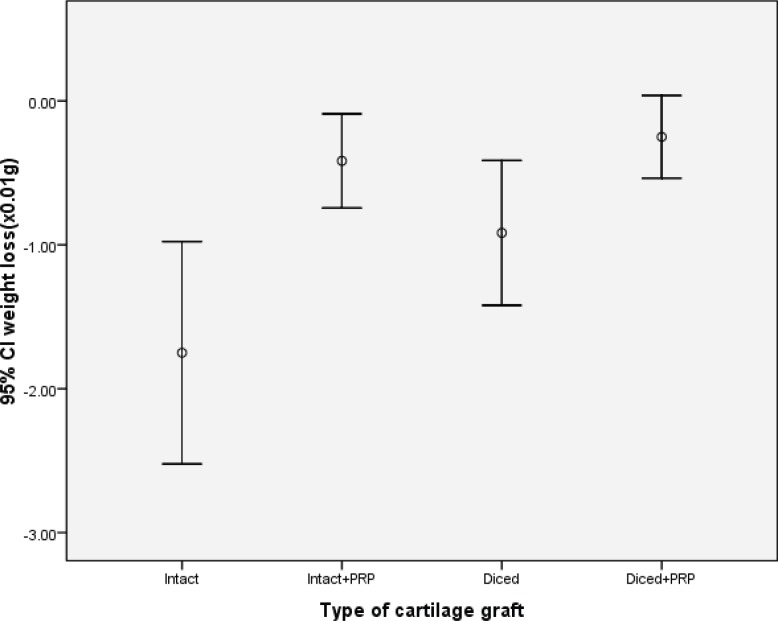
Comparison of the mean weight loss in four different cartilage grafts 12 weeks after implantation.

In the gross examination of the sites of the removed implantations, it seemed that extent of angiogenesis and the diameters of vessels were more pronounced in the side using PRP. The changes of weight and volume of implanted cartilages over the implantation period were illustrated in Table 1. Intact cartilages with PRP had markedly lesser reduction of weight as compared to the intact cartilages without PRP (*p*<0.05; Figure 2). However, this was not true for diced cartilages and the difference of weight loss was inconspicuous between diced cartilages with and without PRP (*p*>0.05).

**Table 1:** Gross and microscopic characteristics of cartilage grafts during a period of 12 weeks implantation.

**Characteristic**	**Intact**	**Intact +PRP**	***P *** **value**	**Diced**	**Diced +PRP**	***P *** **value**
Weight loss	0.0175	0.0042	<0.05*	0.0092	0.0025	>0.05
Volume loss	0.0233	0.0033	<0.05*	0.0083	0.0000	>0.05
Number of nucleated lacunae	27.25	50.50	<0.05*	21.83	42.00	<0.05*

PRP: Platelet-rich plasma. Data were presented as mean.

* Significant

**Fig. 3:**
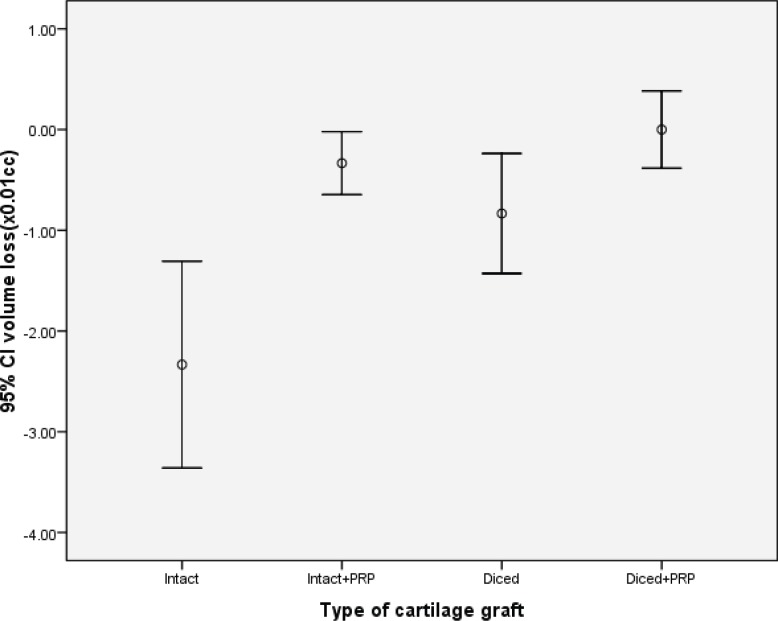
Comparison of the mean volume loss in four different cartilage grafts 12 weeks after implantation.

**Fig. 4:**
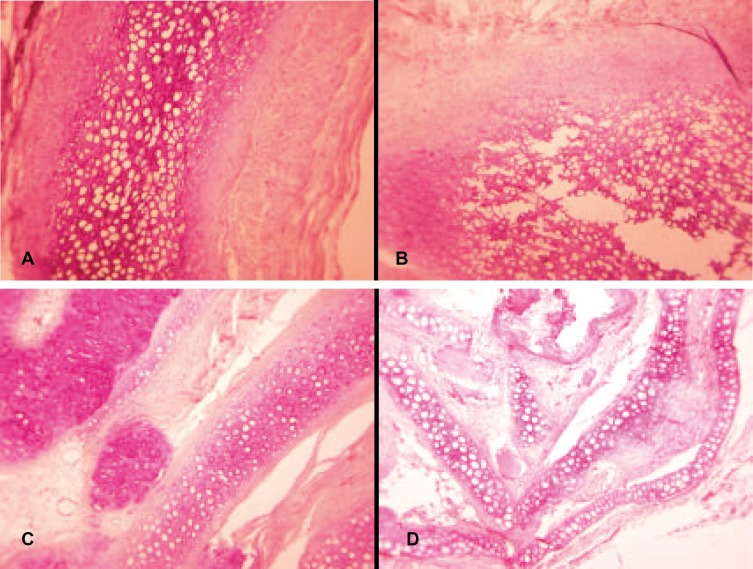
H&E staining of the cartilage grafts 12 weeks after implantation.

**Fig. 5:**
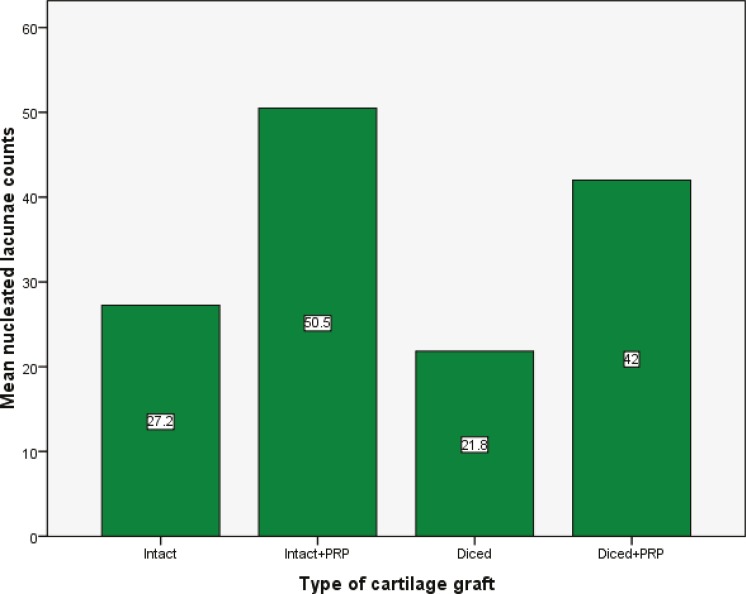
Comparison of the mean numbers of nucleated lacunae in four different cartilage grafts 12 weeks after implantation.

**Fig. 6:**
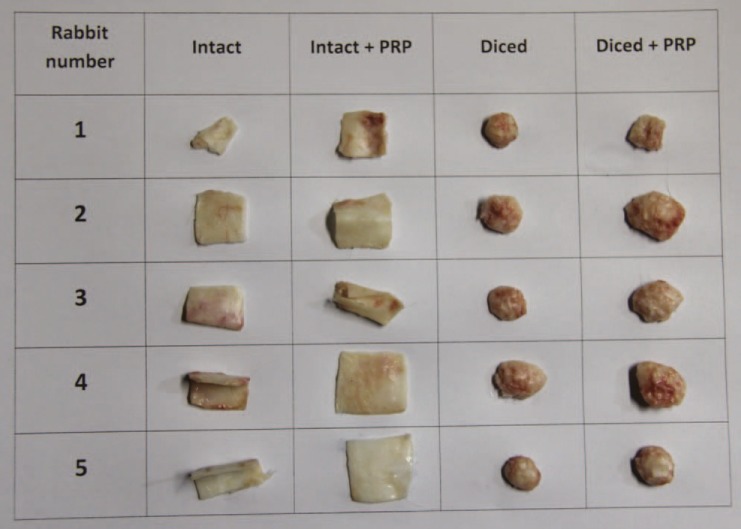
Comparison of the cartilage in 5 rabbits.

Similarly, intact cartilages with PRP had significantly lower volume loss as compared to intact cartilages without PRP (*p*<0.05), while this difference was not statistically significant in diced cartilages (*p*<0.05, Figure 3). Both intact and diced cartilages with PRP had greater number of nucleated lacunae in comparison with those groups without PRP (*p*<0.05; Figures 4-6).

## DISCUSSION

Trauma, malposition and age-related degeneration of articular cartilage are the major causes of cartilage damages. Cartilage has limited potential of self-regeneration and its lesions do not heal spontaneously. Many efforts have been undertaken to support cartilage healing process. Due to lack of vascularization in cartilage tissue, neither inflammation nor the formation of a fibrin clot could help the healing of defects.^14^ The only supportive mechanism is the chondrocytes or synoviocytes of the surrounding tissue which may enhance filling of the defects by increasing their proliferation and matrix synthesis.^14^ As a result, it seems that the most effective protective strategy for healing of cartilage tissue should target these structures.

The main finding of the present study is that adding PRP to intact cartilage grafts has beneficial effects in maintaining their weight and volume. However, this effect was not significant in cases with diced cartilage grafting. In the present study, we assessed the viability of graft cartilages by measuring their weight and volume. The numbers of nucleated lacunae reflect the regeneration capacity of the grafting cartilages.

A growing body of evidence suggests that platelet concentrates might affect cartilage formation through a variety of pathways.^15^-^17^ Mishra *et al.*^18^ in an in vitro investigation reported that PRP enhanced chondrogenic differentiation. In a rabbit model, Saito^19^ showed that administration of gelatin hydrogel micro-spheres containing PRP has preventive effects against osteoarthritis degeneration. Besides these experimental investigations, some current clinical studies support the role of PRP for the treatment of cartilage defects. Kon *et al.*^20^ in a pilot study of 100 patients showed that treatment of cartilage lesions with intra-articular injections of PRP has considerable evidences of safety, pain control and improved function. After two years of follow up, they showed an overall deterioration. Although the persistence of effects widely varied a median duration of 9 months was reported for the beneficial effects. In fact, a greater and longer effect was seen in young men, with a low BMI and a lower degree of cartilage degeneration. More-over, Lee *et al.*^21^ showed that PRP in association of hydrogel could enhance the differentiation of chondrocytes both in vitro and in vivo and thus could help articular cartilage repair.

Although the amount of weight and volume loss in diced cartilages with PRP was less than those of diced cartilages without PRP, this was not statistically significant. However, histologic examination revealed that PRP had conspicuous effects on viability and regeneration of diced cartilages. This encountered discrepancy between gross and microscopic evaluation may be due to the technical errors. Free spaces between the diced cartilages which would be filled by fibro scar tissue may influence their weight and volume measurement.

In the present study none of cases developed infection. As a result, no judgment could be made regarding the effects of PRP on the incidence of infection at the site of cartilage grafting. Moreover, a gross evaluation of grafting sites showed signs of angiogenesis in graft areas which PRP has been used.

However, it is worth noting that using PRP may be associated with some risks in the animal model.^22^ This evidence suggests that more studies in humans should be undertaken before a wide application of PRP in the clinical practice. Little consensus exists in the hematology literature regarding the exact definition of PRP, or its ideal preparation technique. The PRP preparation technique which was used in the present study provided significant concentrates of platelets; with a mean PRP platelet count of ٩۵٠,000/mm3 in comparison with the mean platelet poor plasma (PPP) platelet count of 130,000/mm3; which shows the proper technique of PRP preparation.

In conclusion, our findings yielded valuable information on the effect of PRP on survival and regeneration of autologous cartilage grafts as the extent of angiogenesis and the diameters of vessels were more pronounced in the side using PRP and markedly lesser reduction of weight and volume were visible in this group showing that PRP was effective in increasing survival and regeneration capacity of cartilage grafts in rabbit model. More clinical studies are needed to justify the theoretical benefits of this non-invasive and biological approach.

## CONFLICT OF INTEREST

The authors declare no conflict of interest.
